# Advanced Brain Age Prediction Using Multi-Head Self-Attention: A Comparative Analysis of Western and Middle Eastern MRI Datasets

**DOI:** 10.21203/rs.3.rs-6342594/v1

**Published:** 2025-04-08

**Authors:** Matin Irajpour, Majid Barekatain, Mahdieh Karami, Shaghayegh Karimi Alavijeh, Mohammad Barekatain, Masih Irajpour, for the Alzheimer’s Disease Neuroimaging Initiative

**Affiliations:** 1 Institute for Cognitive Science Studies, ICSS, Tehran, Iran.; 2 Medical Physics and Medical Engineering Department, Medical School, Tehran University of Medical Sciences, Tehran, Iran.; 3 Department of Mathematical Sciences, Sharif University of Technology, Tehran, Iran.; 4 School of Medicine, Isfahan University of Medical Sciences, Isfahan, Iran.

**Keywords:** Brain Age, CNN, Multi-head, Self-Attention, Neuroimaging ethnic differences, Magnetic Resonance Imaging, Neurodegeneration normal aging

## Abstract

Brain age estimation is a critical biomarker for early detection of neurodegenerative diseases, but existing models are primarily trained on Western datasets, limiting their applicability to diverse populations. Recent studies suggest that brain aging patterns vary across ethnic groups, highlighting the need for more inclusive and adaptable AI-driven neuroimaging models. We trained our model on 4,635 healthy individuals (40–80 years) from ADNI, OASIS-3, Cam-CAN, and IXI, using 80% of data (n=3700) for training and 20% (n=935) for testing. The model was further tested on a Middle Eastern dataset (107 subjects, Tehran, Iran). It integrates multi-head self-attention along with residual connections to enhance long-range spatial feature learning, improving upon previous CNN models. Performance was evaluated using mean absolute error (MAE). The model achieved state-of-the-art accuracy (MAE = 1.99 years) on the Western test set, while being much lighter than previous models (approximately 3 million parameters); however, it performed significantly worse on the ME dataset (best MAE = 4.35 years, final = 5.83 years). Bias correction did not improve performance, indicating population-specific brain aging differences. These findings emphasize the need for diverse training datasets and cross-population adaptation techniques.

## 1 Introduction

### 1.1 Background & Importance

Dementia and cognitive decline are among the most pressing public health challenges of our time, affecting millions of people worldwide, with projections indicating a continued rise in prevalence in the future. Catching neurodegenerative diseases like Alzheimer’s disease (AD) and mild cognitive impairment (MCI) early is critical for effective intervention and better disease management [1]. One promising approach in this field is brain age estimation, which predicts a person’s biological brain age based on structural and functional brain changes. A higher estimated brain age compared to an individual’s chronological age may indicate an increased risk of cognitive decline, neurodegeneration, and dementia [2]. Recent studies have already leveraged brain age estimation models to differentiate between cognitively healthy individuals and those with MCI or AD [3–6].

A significant advancement in the development of deep learning models is the introduction of attention mechanism; which has been used extensively in the field of natural language processing and marked a major leap forward for that field. Attention mechanism has also been used for computer vision tasks but not as extensively. Multi-head self-attention mechanisms, commonly used in transformer models, allow models to capture complex spatial dependencies in neuroimaging data more effectively than traditional convolutional networks. These attention-based models have been used in neuroimaging field but they are not widely adopted [7, 8].

### 1.2 Limitations of Existing Models

Despite the success of recent brain age models, most have been trained on Western datasets, often relying on large-scale public neuroimaging resources such as the UK Biobank, IXI, OASIS, etc. [9, 10]. While these datasets have facilitated breakthroughs in brain age prediction, the fact that they primarily include cases from US, UK and Canada, leads to a critical limitation: brain aging is not a universal process; it is influenced by genetic and environmental factors. Ethnic and regional differences in diet, lifestyle, socioeconomic status, healthcare access, and genetic predisposition contribute to distinct brain aging trajectories across different populations [11, 12]. Other studies, focusing on structural changes have also substantiated this hypothesis, different ethnic populations show different patterns of brain aging [13, 14]. This variability has important implications, a model trained solely on Western datasets may fail to generalize effectively to non-Western cohorts, leading to biased predictions and reduced accuracy. Also, accounting for these differences is crucial for developing personalized care plans [15].

The Middle Eastern population remains largely underrepresented in brain age research, despite being home to a diverse genetic pool and unique environmental influences. To date, no large-scale neuroimaging dataset from Middle Eastern subjects has been integrated into standard brain age models, limiting our understanding of how brain aging differs across this populations. Addressing this gap is crucial for improving model generalizability and ensuring that brain age estimation can be applied effectively across diverse populations.

### 1.3 Study Objective & Contributions

This study aims to bridge the gap in brain age estimation research by developing a state-of-the-art multi-head self-attention model and evaluating its performance across both Western and Middle Eastern populations. Our key contributions include:

1. Development of a novel Brain Age estimation model
  - We introduce a lightweight deep learning model incorporating multi-head self-attention to enhance feature extraction and performance, which outperforms prior brain-age models.
  - The model is trained and validated on a number of large Western datasets to establish baseline performance.
2. Cross-Cultural Comparison of Brain Aging Patterns
  - We test the model on a novel Middle Eastern neuroimaging dataset to assess its generalizability.
  - Statistical analyses are conducted to determine potential differences in brain aging process between the datasets
3. Identification of Model Bias & Limitations
  - We analyze the model’s error distribution across datasets to quantify any performance disparities.
  - We discuss the implications of using Western-trained models on non-Western populations and propose strategies for improving fairness and robustness in brain age prediction

By investigating these aspects, this study contributes to a more inclusive and generalizable approach to brain age estimation, paving the way for improved early diagnosis and intervention strategies in neurodegenerative diseases across diverse populations.

## 2 Results

### 2.1 Dataset Characteristics

The datasets used in this study included a large Western cohort for training and testing, as well as an independent Middle Eastern dataset for external validation. Table 1 summarizes the mean age and standard deviation for each dataset. The age distributions across the datasets reflect a balanced representation of middle-aged and older adults, ensuring that the model was trained and tested on a diverse range of brain ages.

### 2.2 Model Training Performance

**Fig. 1:**
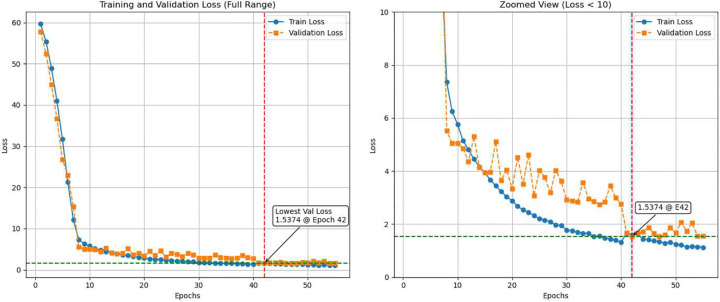
Training and validation loss across epochs

The proposed model was trained on the Western dataset using an 80*/*20 train-validation split. Figure 1 illustrates the training and validation losses across 55 epochs, showing a steady decrease in error as the model converges. The validation loss closely follows the training loss, suggesting that the model generalizes well to unseen data during training without significant overfitting.

### 2.3 Model Performance on Test and Middle Eastern Datasets

**Fig. 2:**
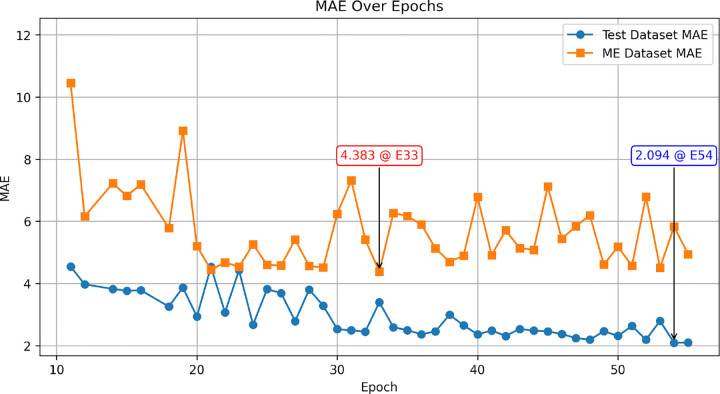
Performance of the proposed model on test set and Middle Eastern dataset across epochs

To assess how well the model generalizes across different populations, we evaluated its performance on both the Western test set (935 subjects) and the Middle Eastern (ME) dataset (107 subjects). Figure 2 illustrates the mean absolute error (MAE) across epochs for both datasets.

The model achieved a final MAE of 2.09 years on the Western test set, achieving state-of-the-art performance and indicating strong predictive accuracy within this population. However, performance on the Middle Eastern dataset showed noticeable variation depending on the training epoch. Notably, checkpoints that performed better on the Western test set often performed worse on the ME dataset. For example, at epoch 54, the model achieved one of its lowest MAEs on the Western test set (2.09 years) but exhibited a significantly higher MAE on the ME dataset (5.83 years). Conversely, the lowest MAE recorded for the ME dataset was 4.35 years at epoch 33, a checkpoint where Western test performance was worse than its previous and next epochs. These findings suggest potential population-specific differences in brain aging patterns.

### 2.4 Effect of Bias Correction

Brain age models often exhibit systematic bias, where predictions tend to deviate from chronological age in a consistent manner, usually overestimating the brain age. Beheshti et al. [17] proposed a post-hoc correction method using a linear regression model, where predicted age (*X*) is mapped to chronological age (*Y*) in the form of *Y* = *aX* + *b*. By fitting this equation on the training set, the slope (*a*) and intercept (*b*) can be calculated and then applied to adjust predictions on the test set, improving model accuracy.

Following this approach, we applied bias correction using a linear regression model trained on the Western training set. This correction was then applied to both the Western test set and the Middle Eastern dataset to assess its impact. Table 2 presents the MAE before and after bias correction. Calculated slope and intercept were 0.9701 and 2.114 respectively. Interestingly, the correction model based on Western train set increased the error rate on the ME dataset. Implications of this finding will be discussed at length in the discussion section.

### 2.5 Comparison with Existing Brain Age Models

To contextualize our model’s performance, we compared its results with similar studies in brain age prediction using deep learning-based architectures. Table 3 presents a comparison of MAE values reported in previous studies. Our model achieved state-of-the-art performance on multiple datasets, while keeping the parameter count low (≈ 3 million), making it computationally efficient.

## 3 Discussion

### 3.1 Summary of Findings

This study introduced a novel 3D CNN with multi-head self-attention for brain age estimation and assessed its performance on both Western and Middle Eastern populations. The model achieved state-of-the-art accuracy on the Western test set, with a mean absolute error (MAE) of 1.99 years, outperforming previous deep learning-based models; while being efficient and lightweight, without resorting to computationally expensive techniques such as ensemble learning or segmentation. However, when tested on the Middle Eastern dataset, the model exhibited a noticeable drop in performance, with the lowest MAE recorded at 4.35 years and an MAE of 5.83 years on the epoch that performs best on Western dataset. These results indicate that while the proposed model excels in predicting brain age within the Western population, its performance degrades heavily when applied to non-Western populations, highlighting potential population-specific differences in brain aging patterns.

Interestingly, the application of post-hoc bias correction worsened the predicted ages for the ME dataset. This finding underlines the potential different ageing patterns in Middle-eastern populations compared to Western populations; indicating the need for diverse and representative neuroimaging datasets to ensure robust and equitable brain age prediction models. A large dataset of Middle Eastern subjects is essential for development of a brain age model for this population.

### 3.2 Comparison with Previous Brain Age Models

Brain age estimation models based on structural MRI data generally fall into two categories: traditional machine learning models that rely on morphometric feature extraction, and deep learning models that process MRI images and automatically extract relevant features using convolutional layers. Over the past few years, deep learning approaches have consistently outperformed traditional machine learning models in terms of mean absolute error (MAE), primarily due to their ability to learn complex, high-dimensional features directly from imaging data.

One of the most notable deep learning models in this field is the Simple Fully Convolutional Network proposed by Peng et al. [9], which achieved a state-of-the-art MAE of 2.14 years on the full UK Biobank dataset (n=14503). They also proposed an ensemble learning approach for a smaller sized dataset (n=2590), which achieved similar performance but at the cost of significant computation cost. This method required training five separate models for each of four different types of preprocessed images (linearly registered, non-linearly registered, grey matter segments, and white matter segments). The predictions from these 20 models, were then combined using ensemble learning, which further increased the computational demands. Both of their models were trained on a restricted age range (42–82 years) in the UK Biobank dataset, limiting its applicability to younger and older populations. When trained and tested on a more diverse dataset (PAC-2019, covering ages 14–94), the model’s performance degraded, yielding a higher MAE of 2.9 years compared to 2.14 years for UK Biobank.

Our proposed model achieves better performance on a dataset with a comparable age range to UK Biobank (40–80 years) and similar size to the second approach, without the added complexity of multiple preprocessing pipelines or ensemble learning. Unlike Peng et al.’s ensemble learning approach, our model maintains computational efficiency while integrating multi-head self-attention, allowing it to retain a low parameter count (≈ 3 million) while outperforming both models. This efficiency is particularly important for clinical applications, where computational constraints often limit the feasibility of large-scale deployment.

Beyond accuracy and computational efficiency, generalizability remains a major challenge for brain age models. Many previous studies have been trained exclusively on Western populations, limiting their applicability to more diverse global cohorts. Some strides have been made to address this issue. For example, Dartora et al. [16] trained a 3D CNN on a multi-cohort dataset that included UK Biobank, ADNI, AIBL, GENIC, J-ADNI, and AddNeuroMed, incorporating data from Japanese and Canary Islands populations. While this represents an improvement in dataset diversity, our findings provide direct empirical evidence that Western-trained models do not generalize well to Middle Eastern subjects, reinforcing concerns raised by Thottupattu [13, 14] about ethnic and genetic variations in brain aging patterns.

These results suggest that current brain age models remain population-dependent and that future research should focus on integrating more diverse datasets and developing adaptation strategies to improve cross-population generalizability. Our model demonstrates that multi-head self-attention offers an efficient and scalable approach for brain age estimation.

### 3.3 Potential Explanations for Population-Specific Performance Differences

The significant drop in performance when testing on the Middle Eastern dataset suggests that brain aging patterns may differ across ethnic groups due to multiple factors. First, racial and ethnic background, affects the brain ageing process; resulting in significant differences in volumetric analysis of Caucasian and Korean subjects [11]. In another study, it was concluded that Black middle-aged subjects had an accelerated pattern of brain ageing, compared to Latinx and White adults [12, 15]. Since most brain age models are trained on Western participants, they may fail to capture subtle structural differences present in Middle Eastern populations.

Second, environmental and lifestyle factors likely contribute to distinct brain aging patterns. Research has shown that factors such as diet, physical activity, cardiovascular health, and pollution exposure influence neurodegeneration and cognitive decline [24–26]. Dietary differences, such as higher consumption of omega-3 fatty acids in Mediterranean diets, have been associated with slower brain aging, while increased pollution exposure in urban environments has been linked to accelerated cortical atrophy [27, 28]. These factors could partially explain why a model trained on Western datasets struggles to accurately predict brain age in Middle Eastern subjects. Overall, these limitations underscore the need for more ethnically diverse neuroimaging datasets to improve the fairness and accuracy of brain age prediction models.

### 3.4 Implications for Brain Age Modeling and Clinical Applications

The results of this study have several important implications for the future of AI-driven neuroimaging research and clinical applications. First, our novel deep learning model demonstrates strong predictive performance while maintaining computational efficiency, making it suitable for both research and clinical applications. Unlike many previous models, which require ensemble learning or extensive preprocessing pipelines, our approach directly processes raw MRI images using multi-head self-attention, effectively capturing spatial relationships between distant brain regions—a major limitation in traditional CNN-based models. Previous attempts to address this issue often involved stacking multiple complex convolutional layers, leading to large, memory-intensive architectures. In contrast, our model achieves state-of-the-art accuracy with significantly fewer parameters (≈ 3 million), outperforming much larger models while remaining computationally efficient. This efficiency makes it more accessible for clinical deployment, particularly in resource-limited settings.

Second, our findings reinforce the importance of dataset diversity in training robust, generalizable brain age models. The significant drop in performance on the Middle Eastern dataset suggests that current brain age models trained solely on Western datasets may not be suitable for clinical use in non-Western populations without additional adaptation techniques. While Western datasets such as UK Biobank and ADNI are widely used in neuroimaging research, populations from Middle Eastern, African, and Eastern European regions remain underrepresented. The lack of diversity in training data limits the applicability of brain age models and may contribute to biased predictions when applied to new populations. Future research should prioritize expanding neuroimaging datasets to include subjects from diverse ethnic and racial backgrounds, ensuring that models perform equitably across global populations.

Finally, our results highlight the potential for personalized brain age estimation. Instead of applying a one-size-fits-all approach, future models should incorporate demographic, genetic, and environmental variables to provide more precise, population-specific predictions. A personalized approach to brain age estimation could help detect early signs of neurodegeneration more accurately, improving individualized risk assessments for Alzheimer’s disease, mild cognitive impairment, and other age-related disorders. Personalized models could also be used to monitor brain aging trajectories over time, enabling early interventions for individuals at risk of accelerated cognitive decline.

### 3.5 Limitations and Future Work

Despite its contributions, this study has some limitations. The Middle Eastern dataset (*N* = 107), while valuable, is relatively small compared to large-scale Western cohorts; therefore, it was only used for testing purposes. Future studies should include larger and more diverse Middle Eastern samples suitable for training models, as well as testing. Additionally, our study relied solely on MRI-based structural features; incorporating multimodal biomarkers such as genetic risk scores and functional MRI data could improve prediction accuracy. Furthermore, this study used cross-sectional data, meaning that conclusions about aging trajectories are based on single-timepoint observations. Longitudinal studies are needed to track individual brain aging patterns over time and confirm whether observed cross-population differences persist longitudinally.

### 3.6 Conclusion

This study introduced a multi-head self-attention-based brain age model that achieved state-of-the-art performance (MAE = 1.99 years) on Western test sets while exposing critical generalizability issues when applied to Middle Eastern populations (MAE = 5.83 years). These findings highlight the importance of diverse training datasets and suggest that population-specific differences in brain aging must be accounted for in future models. Moving forward, integrating cross-population datasets, domain adaptation techniques, and personalized learning approaches will be crucial for developing equitable and clinically useful brain age prediction models.

## 4 Methods

### 4.1 Dataset Description

To develop and evaluate our brain age estimation model, we used a combination of publicly available Western neuroimaging datasets and a newly collected Middle Eastern dataset. The Western dataset consisted of four widely used neuroimaging cohorts: the Alzheimer’s Disease Neuroimaging Initiative (ADNI), Open Access Series of Imaging Studies-3 (OASIS-3), Cambridge Centre for Ageing and Neuroscience (Cam-Can), and Information eXtraction from Images (IXI). A total of 4,635 healthy individuals aged 40 to 80 were included, 80% used for training (3,700 subjects) and 20% for testing (935 subjects). To ensure consistency, only 3T MRI scans were included from ADNI, and all participants with a history of neurological or psychiatric conditions were excluded. The 40 to 80 age range was chosen because major public datasets, such as ADNI, Cam-CAN, and UK Biobank, as well as most previous studies in brain age prediction, have focused on this age range [9, 10, 16]. This selection ensures that our results remain comparable to existing literature while capturing critical aging-related brain changes.

To assess the generalizability of the model beyond Western populations, we gathered an independent Middle Eastern dataset in Tehran, Iran, using a 3T MRI scanner (Siemens, Prisma MRI scanner). The protocol of this study was approved by the Institutional Ethics Committee of Institute for Cognitive Science Studies (ICSS) Tehran, Iran (IR.UT.IRICSS.REC.1403.016). After an adequate explanation of the project to the patients, a written informed consent was obtained before enrollment to the study. Participants underwent cognitive screening with the NuCog test and completed a structured psychiatric questionnaire to rule out cognitive impairment and psychiatric disorders. Also, the MRI images were clinically assessed for any pathologic signs and atrophy by two independent radiologists. Only individuals without any neurologic or psychiatric conditions, with completely normal MRI were included, resulting in a final dataset of 107 healthy participants. This dataset was used exclusively for testing to explore potential differences in brain aging patterns between Western and Middle Eastern populations.

### 4.2 Preprocessing & Data Augmentation

All MRI scans underwent a standardized preprocessing pipeline using SimpleITK (SITK v2.4.0) and Advanced Normalization Tools (ANTs v0.5.3) to ensure consistency across datasets. First, N4 bias field correction was applied using SITK to correct for intensity inhomogeneity, followed by registration to the MNI152 atlas using ANTs with SyN registration, a non-linear registration method, to ensure anatomical alignment across subjects. Brain extraction and skull stripping were performed using ANTsPyNet, and all images were resized to 160×192×160 voxels using ANTs. Finally, intensity normalization was applied using SITK to standardize image intensities across subjects. To minimize the variations introduced by different MRI scanners and acquisition protocols, implementation of a standardized preprocessing pipeline was essential.

**Fig. 3:**
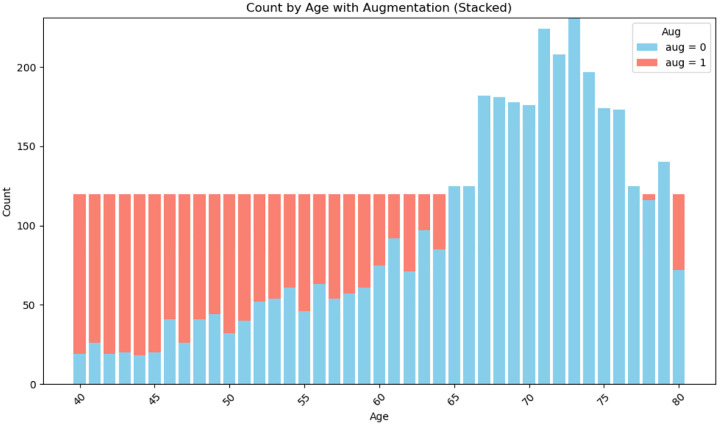
Distribution of cases by age in the training set. Blue bars represent real data, while red bars indicate augmented data.

For improving the model’s robustness and balancing the training dataset, data augmentation was applied to ensure that each age group had at least 120 samples (Figure 3). This included random rotations of up to ±10 degrees and spatial shifts of up to 5 voxels along each axis. The augmented training dataset was then split into 80% training and 20% validation sets. Preprocessing and augmentation scripts are publicly available at Github repository.

### 4.3 Model Architecture & Training

**Fig. 4:**
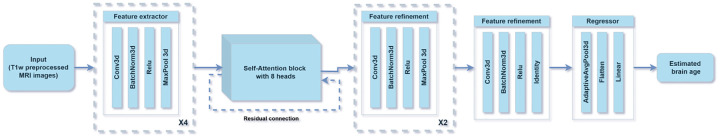
Model Architecture

The proposed model (Figure 4) is designed specifically for processing three-dimensional MRI scans, leveraging a combination of convolutional layers for local feature extraction and a multi-head self-attention mechanism to capture long-range spatial dependencies. The architecture is structured into three main stages: initial feature extraction, attention-based global feature modeling, and final feature refinement leading to prediction.

#### 4.3.1 Feature Extraction with Convolutional Layer

The first stage processes the input 3D MRI volume, which is treated as a single-channel image, through a series of four convolutional layers. Each of these layers continues using 3×3×3 convolutions, batch normalization, ReLU activations, and max pooling to progressively refine spatial hierarchies within the MRI scans. This stage ensures that the model effectively captures local structural features while reducing computational complexity.

#### 4.3.2 Multi-Head Self-Attention for Global Context Learning

After extracting local spatial features, they are passed into a multi-head self-attention block, which enables it to model long-range dependencies within the brain’s structure. In this module, separate 1 × 1 × 1 convolutions are used to generate query, key, and value representations, each mapping the 128 input channels to 256 channels. These feature representations are then split into eight attention heads, allowing the model to compute scaled dot-product attention across different subspaces.

In each attention head, the scaled dot-product attention mechanism is defined as:

$$\text{Attention}(Q,K,V)=\text{softmax}\left(\frac{QK^{T}}{\sqrt{d_{k}}}\right)V$$

where *d*_*k*_ represents the dimensionality of each attention head (i.e., *d*_*k*_ = 256*/*8 = 32). This scaling factor $\sqrt{d_{k}}$ prevents excessively large dot-product values, stabilizing gradients and improving training efficiency.

Each voxel within the 3D MRI scan is thus able to attend to all other voxels, improving the model’s ability to recognize non-local structural relationships and capture complex patterns of brain aging. The outputs from all eight attention heads are concatenated and projected back to 128 channels through an additional 1 × 1 × 1 convolution to ensure dimensional consistency.

To enhance stability and retain the original feature representations, we incorporate a residual connection, where the attention-enhanced features are added element-wise to the original input:

$$\hat{X}=X+\text{MHSA}(X),$$

where *X* represents the original input feature maps, and $\hat{X}$ represents the output after self-attention block. This skip connection ensures that information from earlier layers is preserved, mitigating the risk of vanishing gradients during backpropagation. Finally, layer normalization is applied to maintain a stable distribution of activations, improving convergence and preventing numerical instability.

#### 4.3.3 Feature Refinement and Prediction

Following the attention block, the model further refines its learned representations through three additional convolutional layers; using a 3 × 3 × 3 convolution, batch normalization, ReLU activation, and max pooling. Unlike the earlier blocks, this last stage avoids max pooling, instead employing an identity operation to preserve spatial resolution and retain fine-grained information necessary for accurate age prediction.

At the final stage, an adaptive average pooling layer compresses the 3D feature maps into a 1 × 1 × 1 representation per channel, effectively summarizing the global information present in the MRI scan. Afterwards, the pooled features are flattened into a one-dimensional vector and fed into a fully connected layer, which produces a single scalar output representing the predicted brain age. The total number of parameters in the model is approximately 3 million, making it considerably more efficient than many deep learning models introduced for this task.

#### 4.3.4 Training and Generalization Strategy

The model was trained using the Adam optimizer with a learning rate of 1e-4 and a batch size of 5. Training was conducted for 55 epochs on a high-performance desktop equipped with an NVIDIA RTX 4090 GPU (24GB VRAM), an Intel i5-12400F processor, and 16GB of RAM. The Western dataset was split into 80% training (3,700 subjects) and 20% testing (935 subjects), with the augmented training set further divided into 80% training and 20% validation. Python 3.10 and Pytorch 2.3.1 were used for development of the model. The Middle Eastern dataset was used exclusively for testing, allowing for a rigorous evaluation of how well the model generalizes to populations beyond those represented in the training data.

#### 4.3.5 Model Advantages

By combining local feature extraction through convolutional layers with global feature modeling via multi-head self-attention, this model is well-suited for analyzing the complex structure of 3D MRI brain scans. The self-attention mechanism compensates for the inherent limitations of convolutional layers by enabling the model to consider spatial relationships across distant regions of the brain. As a result, the proposed architecture effectively captures both fine-grained local anatomical details and global structural patterns, making it a powerful tool for brain age estimation across diverse populations.

### 4.4 Bias Correction

Brain age models often exhibit systematic biases, mostly overestimating the subjects’ brain age. To address this, Beheshti et al. [17] proposed a post-hoc bias correction step using a linear regression model. In this model, predicted brain age served as the independent variable (*X*), while actual age served as the dependent variable (*Y*). The resulting regression equation was then used to correct the predicted brain ages for both the Western test set and the Middle Eastern dataset. This adjustment aimed to mitigate systematic overestimation introduced by the model.

### 4.5 Model Evaluation & Statistical Analysis

To assess the accuracy of the model, we used mean absolute error (MAE) as the primary evaluation metric, which quantifies the average absolute difference between predicted and actual brain age. Shapiro Wilk test was used to assess the normality of age and predicted age across all datasets. Due to the non-normal distribution of age and predicted age, the Mann-Whitney U test was used to compare potential differences between the train and test sets, as well as before and after bias correction. All statistical operations were performed using Scikit Learn v1.6.1, SciPy v1.12.0 and Python version 3.10.14.

## Figures and Tables

**Table 1:** Age distribution of datasets used in the present study

Dataset	N	Age						
		mean	std	min	25%	50%	75%	max
**Western datasets**								
ADNI	2189	71.94	5.09	53	69	72	76	80
OASIS-3	1657	64.60	8.54	42	58	66	71	80
Cam-Can	433	60.39	12.17	40	49	61	71	80
IXI	356	58.37	10.13	40	50	59.5	66	80
**ME dataset**	107	50.31	4.76	40	46	50	54	60

**Table 2:** MAE before and after bias correction on test and Middle Eastern datasets

Dataset	N	MAE before bias correction	MAE after bias correction
**Western datasets**			
ADNI	442	1.24	1.23
OASIS-3	348	1.98	2.00
Cam-Can	82	4.30	4.43
IXI	63	4.23	4.04
**Total**	**935**	**2.09**	**1.99**
**ME dataset**	**107**	**5.83**	**5.96**

**Table 3:** Comparison of our model with similar works

First Author	Year	Model Architecture	Datasets Used	Number of Cases	Best MAE (Years)
**Present study**	**2025**	**3D CNN + Multi-Head Self-Attention**	**ADNI, OASIS, CC, IXI, Middle Eastern Dataset**	**4,742**	**1.99**
Jomsky et al. [18]	2025	VGG-based model with AI-generated cerebral blood volume data	13 datasets including: ADNI, IXI, AIBL, etc.	2,851	3.95
Dartora et al. [16]	2024	3D CNN with 26 layers based on ResNet	UK Biobank, ADNI, AIBL, GENIC, J-ADNI	17,296	2.75
					2.66 (CV)
Condado et al. [14]	2023	Linear regressor (12 features extracted from T1w)	ADNI	629	4.14
Peng et al. [9]	2021	Ensemble of 20 models with SFCN architecture (3D CNN) with segmentation	UK Biobank	14,503	2.14
			PAC 2019	2,638	2.9
Han et al. [19]	2022	Morphometric data + Machine Learning models	HCP	1,113	2.7565
			Cam-Can	601	7.0830
			IXI	567	8.0453
Liu et al. [20]	2022	Multi-feature-based network	Private dataset	2,501	3.73
Hahn et al. [21]	2022	Morphometric features + MCCQR model	German National Cohort, MACS, IXI	30,000	2.94 (test)
					4.57 (IXI)
Bashyam et al. [22]	2020	DeepBrainNet, 2D ResNet-based CNN with ImageNet	UK Biobank, ADNI, etc.	14,468	3.702
Cole et al. [23]	2017	3D CNN with segmentation	Brain-Age Healthy Control dataset	2,001	4.16

Abbreviations: IXI: Information eXtraction from Images, ADNI: Alzheimer’s Disease Neuroimaging Initiative, AIBL: Australian Imaging Biomarkers and Lifestyle Study of Ageing, GENIC: Grupo de Estudios Neuropsicologicos de las Islas Canarias, J-ADNI: Japanese ADNI, HCP: Human Connectome Project, Cam-Can: Cambridge Centre for Ageing Neuroscience, MACS: Marburg-Münster Affective Disorders Cohort Study,

## Data Availability

The Western dataset used in this study was compiled from several reputable sources, including: **Alzheimer’s Disease Neuroimaging Initiative (ADNI) database:** Portions of the data used in this article were obtained from the ADNI database (http://adni.loni.usc.edu/). Investigators within ADNI contributed to the design and implementation of ADNI and/or provided data but did not participate in the analysis or writing of this report. A complete listing of ADNI investigators can be found at: http://adni.loni.usc.edu/wp-content/uploads/how_to_apply/ADNI_Acknowledgement_List.pdf. **Cambridge Centre for Ageing Neuroscience (Cam-Can)** [29, 30] **Information eXtraction from Images (IXI)** [31] **The Open Access Series of Imaging Studies-3 (OASIS-3)** [32] The Middle-Eastern dataset is available upon reasonable request from corresponding author.
